# Maternal Malnutrition and Elevated Disease Risk in Offspring

**DOI:** 10.3390/nu16162614

**Published:** 2024-08-08

**Authors:** Kent L. Thornburg, Amy M. Valent

**Affiliations:** 1 OHSU Bob and Charlee Moore Institute for Nutrition and Wellness, School of Medicine, Oregon Health & Science University, Portland, OR 97239, USA; valent@ohsu.edu; 2 Center for Developmental Health, Knight Cardiovascular Institute, School of Medicine, Oregon Health & Science University, Portland, OR 97239, USA; 3 Department of Medicine, School of Medicine, Oregon Health & Science University, Portland, OR 97239, USA; 4 Department of Obstetrics & Gynecology, School of Medicine, Oregon Health & Science University, Portland, OR 97239, USA

**Keywords:** chronic disease epidemic, epigenetics, maternal nutrition, embryo development, fetal development, developmental origins of health and disease

## Abstract

US populations have seen dramatic increases in the prevalence of chronic disease over the past three generations. Rapid increases in type 2 diabetes and obesity have occurred in all the states but have been particularly striking in the Deep South. These increases have contributed to decreases in life expectancy and to painful elevations in health care costs. The causes of worsening population health are complex and incompletely understood. However, there is strong evidence that vulnerability to chronic conditions is determined in early life. Most chronic diseases are developmentally driven. There are specific stressors experienced in early life that influence epigenetic and structural changes during development. These include malnutrition, severe levels of social stress, toxic chemicals, and low oxygen levels. Most US populations have experienced a decrease in the quality of the food they consume as industrial foods have replaced garden-grown foods. Thus, the consumption of too few nutrients before and during pregnancy and during lactation influences the growth of the placenta and fetal organs and their level of resilience when faced with stresses in postnatal life and particularly as adults. Animal studies have shown that the effects of poor nutrition can be passed on to future generations. The most powerful way that the current epidemics of obesity and insulin resistance can be reversed is by providing key nutrients to prospective mothers and those already pregnant.

## 1. Introduction

Most people automatically assume that providing a healthy diet for mothers is highly important for their growing fetuses. However, the contributions of maternal nutrition in powering fetal growth and development have not been driven by strong scientific evidence until recent decades. A recent surge in clinical studies is providing a new understanding of the benefits of healthy food consumption for everyone. It is becoming increasingly appreciated that eating whole foods and balancing macronutrients during pregnancy is required for normal fetal development [1,2,3].

Animal studies have been crucial in understanding general biological properties of perinatal nutrition in mammals. Many foundational principles of nutrition during reproduction have resulted from extensive animal studies [4,5], some of which can be applied to humans. It is likely that we know more about the nutritional requirements of reproducing farm animals than we do about reproducing people. However, the nutritional requirements for animals during reproduction are species-dependent and can differ greatly from human requirements. Therefore, more investigation is needed to understand human nutrition during the periconception period and its impacts on generational health outcomes.

The unique nutritional requirements of pregnant people are complex considering that the consumption of nutrients before pregnancy, during pregnancy, during lactation, and through infancy and early childhood are all important in determining the health outcomes of the pregnancy [2,3,6]. In addition, people come into pregnancy with different disease conditions or develop pregnancy complications that require individualized nutritional management plans. Because of the varying availability of food, food diversity, and cultures that influence eating patterns around the world, this article provides current information about the relationship between maternal nutrition and lifelong risks for chronic disease among offspring.

## 2. Epidemic of Chronic Disease

According to the Cambridge Dictionary, an epidemic is defined as “the appearance of a particular disease in a very large number of people during the same period of time [7]”. The sudden rise in obesity and diabetes represents an epidemic of chronic disease that has arisen across the last three generations, a very short time in terms of human civilization. There is probably no previous time in human history when such a large global population has been afflicted by a lethal epidemic not caused by an infectious agent. Even the total global mortality associated with the COVID-19 pandemic, with some 3 million deaths [8], pales in comparison to the current 41 million annual global deaths due to non-communicable diseases [9]. In the USA, the Centers for Disease Control and Prevention estimates that 60% of the population has at least one chronic disease and 40% has more than one. This has been reported by The Johns Hopkins University [10]. These include heart disease, cancer, chronic lung disease, stroke, Alzheimer’s, diabetes, and chronic kidney disease. Note that the percentages would be even higher if obesity and chronic sleep disorders, now recognized diseases, were included.

Increasing disease rates are driving total U.S. health care expenditures year after year. Health care expenditures in the US increased by 4.1 percent in 2022, reaching a total of USD 4.5 trillion or USD 13,493 per person, as reported in 2023 [11]. It is unlikely that the US population will long afford the health care-related costs that loom on the horizon at the current rates of increasing disease prevalence. The AHA estimates a doubling of health care costs related to cardiovascular disease from a baseline of USD 500 billion in 2017 to over USD 1 trillion by 2035. **The US leads the world in the expense of medical care while simultaneously leading the world in declining health**. The medical system in the US is focused on treating disease conditions and little on root causes. Thus, because of the suffering of the disease-ridden US population and the unsustainable increases in future costs of health care, the worsening health of the US population has become an urgent matter that must be addressed by national, state, and local governments and non-profit organizations.

**Figure 1:**
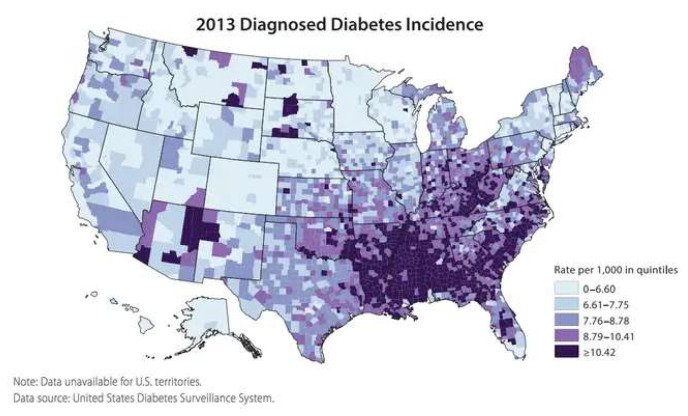
Showing map of the USA and the regional prevalence of diagnosed diabetes in 2013 [12].

There are many reasons cited for the increase in poor health within the US population. Several primary causes have been proposed. The wear and tear theory proposes that people are the products of their own poor lifestyles, the genetic theory suggests that chronic diseases arise from the genes acquired from parents, and the aging theory purports that humans have a built-in clock that underlies the acquisition of chronic diseases as they age. Each of these theories has some validity yet is oversimplified as they do not fully incorporate the ever-expanding evidence of the effects of adverse environments or structural and social determinants on human health. In addition, they do not explain several features of the demographics and the epidemiology of chronic conditions in the US, including the following: (1) the majority of chronic disease cases are found in the southern states (Figure 1) [12]; (2) the rapid increase in the incidence of chronic disease cannot be accounted for by spontaneous increases in gene mutations; and (3) the lack of evidence to support the causality of chronic disease and chronological age alone.

It would be difficult to make the case that severely affected populations have gene variants that lead to disease more commonly than people in other parts of the country. The recent rapid rise in chronic disease incidence and prevalence in the US cannot be accounted for by spontaneous increases in gene mutations that underlie each and every chronic disease, especially for those with high prevalences in the South. Even though genome-wide studies have identified important gene variants associated with many chronic conditions, the prevalence of variants alone does not yet explain large regional hotspots for different diseases [13]. In our current state of ignorance regarding gene interactions and chronic disease, future research may shed more light on these and newly discovered variants that work in tandem to explain some aspects of regional disease. In addition, there is no evidence suggesting that chronic disease is inevitable [14] as a function of chronological age even though the aging process is associated with weakened physiological systems and associated with the activity of the epigenetic clock [15], which is influenced by inherited and environmental stress factors. The various underlying causes of chronic disease will need to be integrated with new knowledge of the epigenetic drivers of disease as they are discovered.

One chronic disease, type 2 diabetes (T2D), is an example of a chronic disease that serves as a “canary in the coal mine”. Its prevalence has been tracked since the late 1950s and is associated with a number of chronic conditions. In the late 1950s, some 1½% of the population was diagnosed with T2D compared to an estimated 11–13% of the population today [16,17]. The rise in the prevalence of overweight and obesity has paralleled T2D prevalence. These conditions are significant contributors to the chronic disease epidemic and are associated with the rise in T2D. Gestational diabetes (GDM) is a condition defined as hyperglycemia identified during pregnancy that is associated with adverse pregnancy and neonatal outcomes and is a risk factor for early progression to T2D during the pregnant person’s lifetime. There is a significant need for more research to understand this form of diabetes. Following a healthy diet is especially important for pregnant people suffering from this malady; they should be under the care of a dietician. The American Heart Association suggests that nearly 67% of the people with diagnosed diabetes die from cardiovascular disease [18]. What has not been addressed by the leaders of the national medical community are the sociological and industrial underpinnings of the vastly increased vulnerability of the population to experience a chronic disease across their lifespan. This vulnerability appears to have increased with each successive generation for the last four generations.

## 3. Chronic Disease Begins in the Womb

**Figure 2:**
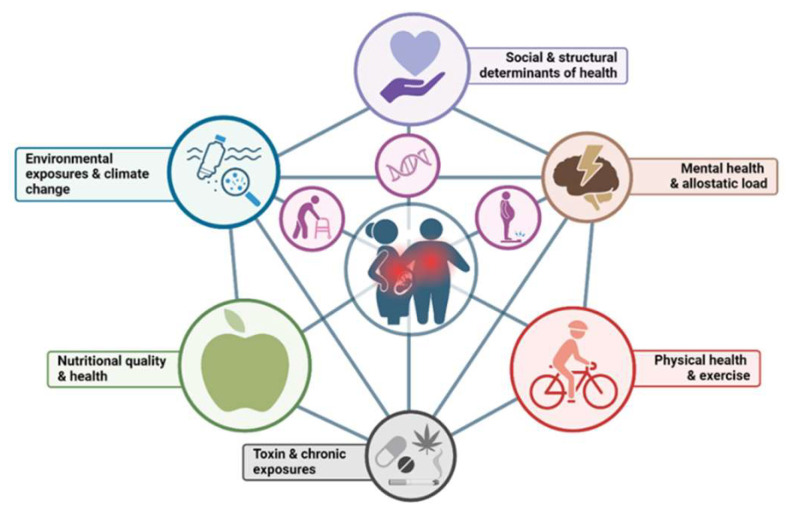
Complex interactions that contribute to chronic diseases that go beyond simply aging, genetics, and “wear and tear” concepts (images represented as magenta). Created with BioRender.com.

The social determinants of health are important influencers of human physiology. Populations with healthy lifestyles are more protected against disease than the populations who live in high-stress environments (Figure 2). Disease rates are related to behavioral patterns including tobacco, illicit drugs, and excessive alcohol use, which not only accrues significant costs to the individual but also the health care system. Less recognized but more broadly impacting our society’s overall health are decreased physical activity in schools and increases in screen time for both children and adults. Perhaps the most influential reason that Western populations are becoming more vulnerable to disease is the increasing consumption of highly processed and nutrient-deficient foods [19,20,21]. Unfortunately, these exposures not only detrimentally affect children and adults but they also increase the susceptibility for disease in people starting from conception in the womb. The consumption of these foods is detrimental to the health of people of all ages. What many health officials have not recognized is that there is increasing evidence that maternal stressors in early life are the culprits causing increasing susceptibility to diseases in offspring. The processed food revolution has augmented susceptibility to health disorders originating in the womb and in the early stages of postnatal life. Highly processed foods promote an inflammatory milieu within organs and increase stress at the cellular level which interferes with normal developmental processes [22] for the embryo and fetus. It also increases the risk of maternal diseases. Malnutrition in the form commonly seen in Western populations is represented by the term, “**high calorie malnutrition**”, where calories are consumed in excess of metabolic needs without accompanying nutrients [23]. This is a common eating pattern for people in the USA and is spreading across the globe.

The Developmental Origins of Health and Disease (DOHaD) is based on a large body of solid evidence showing that the in utero environmental influences of the early stage of development as the underlying cause of adult disease vulnerability. Offspring are impacted by exposures before conception and throughout pregnancy, lactation, and early childhood. However, in order to understand how nutrients positively or negatively affect developmental patterns, it is important to consider the nutritional needs of the embryo and fetus to provide for their optimal development.

Embryonic and fetal development are characterized by sequential biological stages that reflect the sequential processes involved in the manufacturing of organs. The embryonic period is defined as the time when all the organs of the body begin to emerge in their most primitive form. In humans, primitive organ formation (organogenesis) occurs during the embryonic period (weeks 3–8). Each embryonic organ follows its own individual developmental timeline that continues through the fetal period (week 9 to birth [24]). For example, the human embryonic heart begins beating at the end of the 3rd week postconception, during the embryonic period. It beats continuously while remodeling itself [25]. In contrast, the embryonic and fetal lungs are very primitive and not prepared for gas exchange until late in gestation [26]. As embryonic development transitions to the early fetal stages, the primitive organs begin their maturation process. Some are functional relatively early (like the primitive kidney filtering system and the beating heart) but most become functional closer to term.

Even at the early stages of development, organs have different metabolic needs and nutritional requirements to support the necessary cell growth and differentiation needed for proper development [27]. The nutritional status of a pregnant person even prior to pregnancy is critical for nutrient availability for the developing fetus. Nutrient availability (excess or deficient) and disruptors (i.e., stress, inflammation, toxins, etc.) can influence the metabolic pathways and metabolites that are induced and influence the epigenetic control of development. As the embryo and fetus develop, each organ requires a unique combination of biological molecules required for structural formation and biochemical reactions. Many of the micronutrients needed during periods of gestation have been identified [28]. These include choline, folic acid, iron, calcium, vitamin D, omega-3 fatty acids, B vitamins, and vitamin C. The early embryo first acquires these nutrients from uterine secretions and later from maternal blood via the placenta. During the embryonic period before entry into the intrauterine cavity, the early embryo is dependent on pyruvate or lactate metabolism which then switches to dependency on glucose metabolism during the implantation phase of embryonic development, which is important for directing cell differentiation and fate. Vibrant placental transport is required to maintain a robust growth pattern in developing offspring. Embryos and fetuses have so-called critical periods [29] when their cells are rapidly dividing and consuming nutrients. It is during these critical windows of time when nutritional stressors inflict the most damage by interfering with normal cellular patterning and maturation. Should needed nutrients be in short supply, developing organs will be structurally compromised, requiring adjustments in growth and function. Similarly, if the developing embryo and fetus are exposed to toxic chemicals or maternal cortisol, organ development will be suppressed.

**Figure 3:**
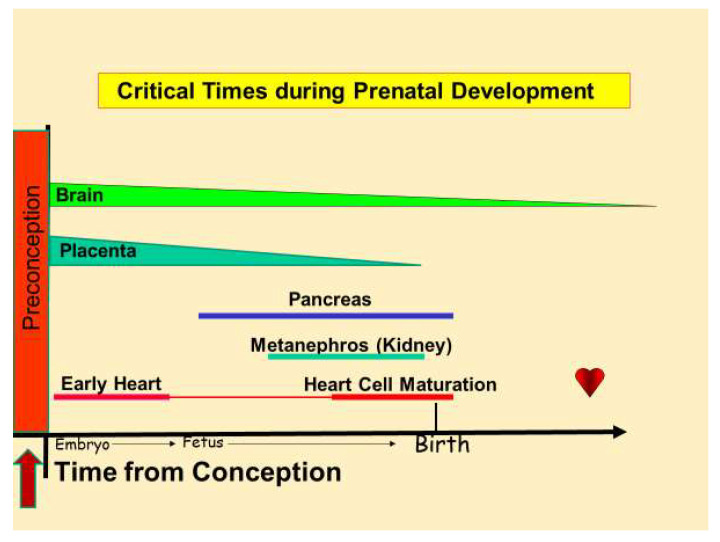
Critical periods of organ development and nutritional vulnerability. The thickness of the bars represents the levels of vulnerability. The lower left arrow denotes the preconception period when the oocyte, sperm, and preimplantation embryo are also susceptible to nutritional insults and epigenetic modification. Nutritional insults include deficits in nutrients in the blood as well as food-derived excesses of substances in the blood [2].

Figure 3 depicts the stages of development during which a few fetal organs are shown to be critically vulnerable to known stressors. The wider the bar, the higher the vulnerability. Because nutritional stress is a common stressor for which the fetus and placenta must accommodate, it is crucial to understand organ-based nutritional needs by the developmental stage when any given organ can be detrimentally affected if malnourished [2]. Stressors affect the fetus in various ways. Each pregnant individual will have varying degrees of chronic conditions, occupational or environmental toxin exposures, allostatic load, and baseline nutritional health coming into the pregnancy. A host of developmental compromises are made possible by the adaptation to stressful environments via mechanisms known as developmental plasticity [30,31]. The adaptation of the embryo/fetus to stress is driven by pleiotropic genes whose responsive plasticity of expression underlies a panoply of changes that ensure viability. However, fetal accommodations themselves may generate risks for disease in later life. For example, if a fetus has limited nutritional resources, the kidney will develop fewer filtering nephrons, which increases the vulnerability for chronic kidney dysfunction in later life [32]. The developing heart is especially vulnerable at two intrauterine stages. In the early stages of organogenesis, the heart undergoes a complex series of structural changes while its cells are proliferating rapidly. The deficits or excesses of nutrients can lead to structural abnormalities [33,34]. Closer to term, the heart goes through a maturation process whereby it prepares for a switch from a mostly fetal carbohydrate “diet” to a free fatty acid one when lipid-rich mother’s milk becomes available after birth [35,36]. Stresses during that stage lead to changes in genes regulating metabolism in the heart [37].

## 4. Linking Maternal Nutritional Stress to Chronic Disease

The prevalence of chronic diseases is increasing worldwide even among children [38]. There is increasing evidence that prenatal accommodations to poor maternal nutrition set the trajectories for a host of chronic conditions including T2D, obesity, stroke, heart disease, as well as chronic obstructive pulmonary disease and liver disease in later life [31,39,40,41]. Eating patterns rich in whole grains, fruits and vegetables, legumes, nuts, healthy plant-based oils, and low-mercury fish are associated with reduced inflammation and better health [42] across all the life stages [43]. However, only a small percentage of Americans actually practice healthy eating patterns [44]. Survey data from the National Health and Nutrition Examination Survey (NHANES) 2011 to 2012 suggested that only 1% of the population was eating a consistently healthy diet whereas over 70% of the population was in the “poor” category. Recent data show some improvement. However, the poor diets in 2009–2010 underlie the nutrition of the people who were pregnant at the time and their associated elevated risks for cardiovascular disease in later life [43].

Eating patterns with higher amounts of red and processed meats, saturated fats, trans fatty acids, added sugars and highly processed industrial food products are proinflammatory and detrimental to health for people of all ages [45]. A recent study in 195 countries points to the inadequate consumption of whole grains, fruits, and vegetables and the excess consumption of salt as the underlying features of chronic disease across the globe and are the primary causes of death. In the US, public health leaders, community leaders, and health care systems will need to prioritize improving maternal health to reverse the chronic disease epidemic. It will not be possible to make sweeping reductions in chronic disease prevalence without directly addressing the problem of poor nutritional health among the people of reproductive age. Human [46,47] and animal [48,49,50] data point to the potential reduction in disease risk in parents and offspring based on sound physiological principles. 

Prolonged maternal hyperglycemia leads to detrimental outcomes in offspring. Table sugar is a disaccharide consisting of a glucose and a fructose molecule combined. Thus, when table sugar is consumed, both fructose and glucose are released into the bloodstream. There are direct links between high concentrations of these sugars in plasma and inflammatory conditions. When glucose levels become elevated, as in the post-prandial levels of glucose in people who have gestational diabetes, advanced glycation end-products (AGEs) are formed [51]. Glycation is a non-enzymatic reaction whereby glucose becomes attached to proteins and other molecules in blood and tissue, which interferes with their normal function. AGES may signal through the AGE-specific receptor, (RAGE), which generates detrimental reactive oxygen species (ROS) and activates inflammatory processes within tissue. Animal studies suggest that ROS interferes with normal placental function leading to adverse outcomes in offspring.

Fructose is metabolized differently than glucose but has equally detrimental effects when concentrations are chronically elevated. High sustained levels of plasma fructose are associated with the release of inflammatory cytokines and increases in triglycerides in the liver, leading to non-alcoholic fatty liver disease. Fetal fructose derives mostly from endogenous sources manufactured from glucose in the placenta [52]. Elevated exogenous fructose from food and drink consumption appears to be involved with a number of metabolic pathways that are detrimental to fetal metabolism and may be an underlying factor in the initiation of preeclampsia [53,54,55]. People born to mothers who had preeclampsia are more likely to die of stroke as adults.

Specific nutrient deficiencies in the fetus are associated with elevated chronic disease risk [56,57] in that individual. The roles of some nutrient deficiencies such as folate, vitamin D, and iodine have been identified in terms of their elevation of chronic disease risk and structural and functional abnormalities including cardiac and neural tube defects. Specific nutrients are important at different stages of organ development and maturation [2,3]. Calcium is required for bone formation, fatty acids are needed for making cell membranes and brain neuron myelination, iron is needed to make hemoglobin in red blood cells, and choline is needed for cell membrane integrity. The most required substance of all for the fetus is water [58] as the net molar flux of water into the fetus is greater than that of any other substance, including oxygen. Water deprivation is associated with adverse fetal/neonatal outcomes. All of the aforementioned substances are used in increasing amounts as gestation proceeds. However, humans do not consume individual nutrients. People consume a variety of foods and only recently has there been a shift to understanding the impacts of food composition on perinatal outcomes. There are perhaps hundreds, if not thousands, of nutrients that are important for the development of all the organs; many have not been adequately studied or, perhaps for some, even identified.

## 5. The Role of the Placenta in Fetal Nutrition

The placenta is an ephemeral organ that transfers nutrients from the pregnant person to the embryo and fetus. It arises from the outer layer of the cells of the free-standing embryo as it implants in the endometrium. The mature human placenta is characterized by a fetal capillary endothelium and a syncytialized chorionic epithelium in direct contact with maternal blood, which is derived from the differentiation and fusion of the metabolically active and non-continuous layer of cellular cytotrophoblast layer [59]. The cytotrophoblast cells metabolize free fatty acids and generate ATP. At its thinnest, the placental barrier consists of syncytiotrophoblast and fetal capillary endothelium. As the sole source of nutrients for the fetus and the provider of hormones and growth factors, the placenta is required for the successful development of the fetus. For this reason, it can also be a culprit in the compromises made during fetal life that lead to chronic disease. This is widely recognized [6,39,60,61,62,63,64]. The placenta is responsible for producing peptide and steroid hormones necessary for sustaining pregnancy. These hormones orchestrate the maternal metabolic adaptations necessary to facilitate the nutritional needs across gestation.

The nutrients in maternal plasma derive from two sources: (1) The rapid turnover of maternal tissues including fatty acids [65] derived from adipose tissue, amino acids from muscle [66,67], and glucose produced in the liver glucose [65]. (2) Maternal nutrient intake with food. Both sources are equally important. Barker made the point that depending entirely on dietary sources for nutrients is a risky strategy for a pregnancy and thus maternal tissue and nutrient stores are also used. However, it would be a mistake to suggest that the relationships between maternal diet and fetal nutrient supply are simple and straightforward. The complex interactions between maternal dietary practices, tissue turnover, and placental uptake are fetal sex-dependent and differ among people who have different conditions of pregnancy, including gestational diabetes and preeclampsia.

The human placenta is composed of fetal-derived tissue and is thus the genetic product of both the mother and the father. It is designed to bring the maternal and fetal bloodstreams into close proximity with just two cell layers separating mother and baby in its thinnest areas. The placenta uses many mechanisms to transport nutrients; these are well described [45,68] and include diffusion, facilitated diffusion, active transport, and endocytosis. There are specific hydrophilic channels that allow the transplacental diffusion of lipid insoluble molecules; the entire surface area of the placenta is available for the diffusion of respiratory gasses. Specific transport systems for amino acids, glucose, and lipids have been identified [69] on both the microvillous and basal membranes of the syncytiotrophoblast and may be altered by disease states [70]. For example, water crosses the placenta under osmotic forces, amino acids by protein transporters, ions by ion transport proteins, and antibodies, lipids, and iron-containing proteins by specific receptors that traverse the syncytiotrophoblast. While it is not clear how all of these mechanisms are integrated, it is clear that there are cellular processes important in coordinating transport which may contribute to fetal compromise under the conditions of maternal stress [71,72]. What is clear is that the placenta plays a crucial role in compromises made by the fetus under stressful conditions and that these lead to disease risk [73]. These abnormal conditions may be associated with non-circular shapes and thickness of the placenta [63].

It is not yet clear how the placenta and the embryo/fetus interact to compromise fetal development because several placental transport systems are compromised with fetal growth restriction [68]. One common outcome of transport systems that are underperforming is preterm birth. The degree to which a diet of low quality in mothers is associated with spontaneous preterm birth remains uncertain [74] though nutrient intake is likely to play a role. Preterm infants, small-for-gestational age (<10th percentile for gestational age), and fetal growth-restricted individuals (intrauterine growth retardation) have elevated risk for chronic disease. Infant size and gestational age at delivery are surrogate markers for the adverse influences of the intrauterine environment on perinatal development. More studies are needed to determine population-based and individualized interventions that can optimize the nutritional health of mother and baby and subsequently placental health and function.

## 6. Epigenetic Drivers of Chronic Disease

Over the past 30 years, the role of epigenetics in regulating gene expression has become mainstream biological dogma. The fact that the chemical modifications of DNA and protein histones along with the roles of non-coding RNA species strongly influence the expression of hosts of genes throughout development suggests an important role in compromises using epigenetic modifications to accommodate fetal stresses. One of the early demonstrations of the influence of nutrition on the epigenetics of offspring comes from experiments by Waterland and Jirtle 2003, [75] using the agouti viable yellow (Avy) isogenic mouse model. They showed that deficits in maternal dietary factors including methyl groups during the pregnancy of the agouti (brown) mouse led to a yellow coat color in offspring accompanied by metabolic dysregulation and a propensity for obesity, hyperinsulinemia, and a shortened lifespan. These effects were directly linked to the degree of the methylation of the Agouti gene which contains a retroviral intracisternal A particle insertion upstream of the Agouti transcription start site. Low degrees of the methylation of the insertion leads to a yellow coat and high levels of methylation results in a healthy brown phenotype. When first published, this direct link of a disease outcome to maternal diet was a revolutionary discovery because it linked maternal diet to offspring outcome. It is now clear that the Avy mouse model can be used to detect a number of maternal stressors that relate directly to the developmental origins of elevated disease risk in later life [76]. A large number of differentially methylated regions of DNA in response to a maternal high-fat diet have been discovered in murine offspring [77,78]. The degree to which methylated regions have enduring effects on gene expression has yet to be determined.

**Figure 4:**
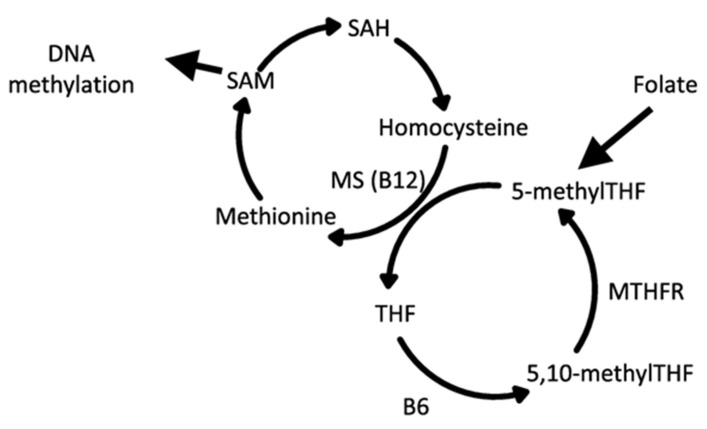
One-carbon metabolic networks that are affected by dietary methyl groups. SAM is the step where methyl groups are made available for DNA methylation [72]. A number of methyl-bearing nutrients are important contributors to this pathway [83]. MS, methionine synthase; MTHFR, methylenetetrahydrofolate reductase; THF, tetrahydrofolate; SAH, S-adenosylhomocysteine; SAM, S-adenosylmethionine.

One important link between dietary nutrients and epigenetic changes is the one-carbon metabolic pathway which integrates metabolic networks with nutrient status and multiple biological actions. The networks generate S-adenosylmethionine (SAM), the universal methyl donor for methylation reactions that underlie histone and DNA methylation [79,80,81]. Figure 4 shows a simplified version of the cycle [82], demonstrating the mechanism in which methionine-rich foods support the generation of methyl groups used in DNA and the histone methylation processes.

Even clear-cut examples in a rodent model that demonstrate intergenerational phenotypic changes do not prove that epigenetic influences persist across multiple generations in human populations. Observational studies suggest that they do. There are increasing numbers of studies reporting transgenerational epigenetic changes [84,85,86] that apply to mammals (Desai et al., 2015) [87]. In one epigenome-wide study, grandmaternal stress during pregnancy led to differential DNA methylation in grandchildren. Five differentially regulated CpG sites (false discovery rate < 0.05) were found in the grandchildren of grandmothers exposed to severe stress while bearing the children’s mothers [88]. The differentially methylated genes were related to processes regulating the circulatory system. Likewise, the children of mothers who suffered nutritional stress during the Dutch Hunger Winter of 1944–1945 were more likely to have elevated disease risks [89] and differentially methylated DNA sites [90] related to a number of genes important for growth and metabolic disease. The grandchildren were more likely to be unhealthy at a younger age than age-matched peers. In spite of increasing circumstantial evidence, there are scientists who remain skeptical [91] of intergenerational epigenetic inheritance. Skepticism is warranted because the field is in its infancy and it is important to ensure that the mechanisms that drive transgenerational phenotypes can be clearly understood in the human population. The role of maternal diet in modifying epigenetic patterns in offspring has been reviewed [92].

There is new attention being paid to the role of hormone-related cancers and neurological sequelae following offspring exposures to maternal stressors. Several studies suggest that elevated estrogens during pregnancy are important in determining the risk for breast and ovarian cancer as well as prostate cancer. These appear to be nutritionally based. It appears that girls born small and grew rapidly as children had higher estrogen levels during early pregnancy, imparting a risk of future cancers [40]. High estrogen during puberty is associated with intercristal hip width and breast cancer in offspring. These growth patterns are related to energy-dense diets during childhood and adolescence and the elevation of the tissue concentrations of estrogen produced by adipose tissue. A recent study [93] in mice showed that a low-protein diet during pregnancy interrupted miRNA-mRNA networks in estrogen production in offspring resulting in the suppressed growth of the prostate in males and an elevated risk of prostate-derived malignancies. Zhu et al. demonstrated that higher consumption of refined grains during pregnancy among pregnant persons with gestational diabetes was associated with higher offspring body mass index z scores and risk of overweight or obesity at age 7. A substitution of one serving per day of refined grains with whole grains correlated with a 10% risk reduction in offspring overweight or obesity by age 7 [94].

In addition to structural changes in organs that occur in response to maternal conditions in the womb, there are additional human examples of epigenetic changes that are associated with poor nutrition [92] in early development. In a study of adiposity among 9-year-old children, it was found that the methylation status of retinoic acid receptor-α was associated with low maternal carbohydrate intake in early pregnancy [95]. It is now possible to link diet quality with age-related diseases [96], some of which are determined during prenatal stages of development.

## 7. Nutrition and the Modern Food Culture

Since the mid-20th Century, the eating habits of people in the USA have become increasingly dependent on industry-supplied food. A sampling of advertisements in the 1950s newspapers, magazines, and later television demonstrates how the food industry has been catering to the basic tastes of everyday people to expand their market share for tasty, addictive foods. Advertisements promoted non-nutritious messaging by suggesting adding 7-Up to baby bottles, urging parents to ensure that teenagers receive enough sugar every day, or eating vitamin B1-enriched doughnuts, all of which were, in reality, harmful nutrition advisories. In 2021, the food industrial complex including agriculture- and food-related industries added USD 1.264 trillion to U.S. gross domestic product (GDP) [97]. Thus, the food industry is an important part of the US economy and is not likely to change course in any dramatic way in the near future.

Now in the early 21st century, only a small percentage of people eat wholesome foods raised on farms. It appears that people have shifted their taste preferences away from freshly prepared fruits and vegetables in favor of highly processed foods and “fast” or nutrient-deficient foods. This shift in the eating habits of US residents has occurred because the industry tailors its products to reach the “bliss point” with the right mixture of salt, sugar, and fat [98] to make food tasty and addictive. It is becoming increasingly evident that highly processed foods are associated with a number of disease conditions [99] in adults but not as well recognized are the transgenerational harms of these foods when exposed during pregnancy. Animal studies have shown long-lasting adverse outcomes related to poor unhealthy diets [100,101,102]. What is not generally appreciated in the medical community is that the greatest harm from such foods is inflicted during early life. As data accumulate in human studies, this aspect of nutrition will become increasingly recognized as it has been by farmers who raise animals for decades [100].

In addition to nutrient-deficient foods on supermarket shelves, there are a number of “fad diets” marketed by so-called experts that are likely to have long-term detrimental effects on most people but especially on offspring exposed during pregnancy. These include fasting, the keto, paleo, and Atkins diets, as well as the grain brain diet. Several of these diets have become popular because they promote weight loss. Even though weight loss can be beneficial for some people on healthy diets, the unintended consequence (i.e., harm in losing weight) of eliminating important macronutrients just before or during pregnancy can contribute to adverse outcomes including structural abnormalities in the fetus [103]. Some weight loss diets can contribute to cardiovascular disease. Those based on questionable metabolic theories are, nevertheless, lucrative for purveyors and publishers without the consideration of the health consequences of unsuspecting consumers. These diets have been reviewed and evaluated by the American Heart Association [104].

While it has been known for many years that a proinflammatory diet is associated with adverse outcomes in offspring, the mechanisms have not been well understood. That state of ignorance is largely the case today, but two new promising areas of research are enlightening the field. (1) It is now clear that alterations in the composition of the maternal microbiome are associated with diets containing high levels of refined carbohydrates, saturated fats, and salt. Ultra-processed foods are associated with cardiometabolic risk [105]. Such diets may compromise placental and fetal health [106]. (2) In addition, there are a host of detrimental cytokines that are increased in the plasma of pregnant people who consume proinflammatory foods. These include the interleukins 4,6,12, and 17 as well as TNFα. These cytokines appear to be related to the methylation status of imprinted genes [107].

High calorie malnutrition leads to excess body fat which is a risk factor for compromised semen quality in men and adverse pregnancy outcomes. Adipose tissues release adipokines including leptin and adiponectin as well as adipocytokines like interleukin 6 and resistin [108]. These each have independent effects on maternal tissues and placenta function. Excess levels of body fat in pregnant individuals can lead to high levels of circulating leptin and associated leptin resistance in the central regulation of appetite. The regulation of adiponectin may be key in regulating maternal glucose levels as well as integrated transport function in the placenta [109].

The goal for dietary eating patterns during pregnancy should focus on optimizing the intrauterine environment to promote normal fetal growth and development. Healthy eating patterns should include consuming a diversity of cultural foods to achieve a balance of macronutrients including carbohydrates, fats, and protein, and contain rich micronutrients and fiber. Reducing the consumption of saturated fats, ultra-processed foods, added sugars, and sodium and replacing them with higher intakes of nutrient-rich foods including vegetables, fruits, whole grains, legumes, nuts, low mercury fish and seafood, monosaturated plant oils (olive oil), and lower fat dairy can promote longevity and health for mother and offspring.

## 8. Epigenetic Reversal

**Figure 5:**
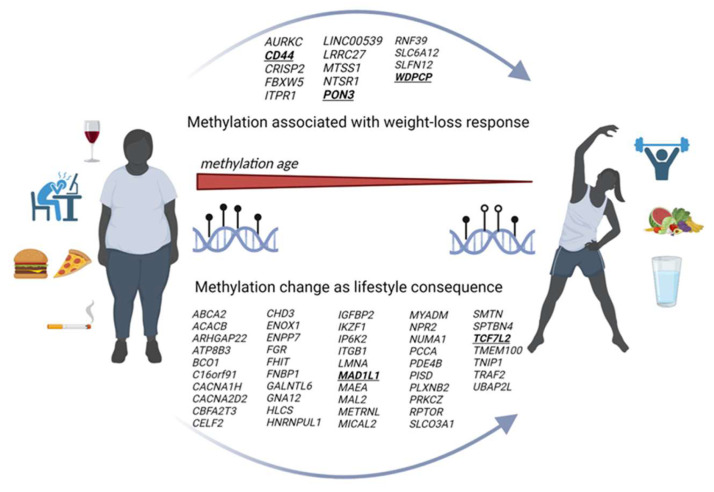
The methylation changes in specific genes associated with improving lifestyle [50].

One of the great mysteries in DOHaD science relates to the degree to which nutrient-deficient dietary patterns during pregnancy induce the epigenetic modifications of genes in offspring that can be reversed after birth. It is known that nearly every improvement in lifestyle patterns for people of all ages has led to modifications in DNA methylation patterns [50]. See Figure 5. More human studies are needed to evaluate epigenetic benefits, though an increasing number of studies have shown beneficial epigenetic modifications during postnatal life [110,111]. If there is any one weakness in our present view of the roles of epigenetics associated with poor maternal diets, it is the fact that we have not established many links between insult and epigenetic modifications including histone remodeling, changes in non-coding RNA and DNA methylation, and propensity for acquiring disease. However, data suggest that even small behavioral changes can lead to long-term benefits. Even among women with a genetic susceptibility or family history of T2D and a history of gestational diabetes, each additional modifiable risk factor (i.e., smoking, weight, nutrition/eating patterns, alcohol intake, or physical activity) that was optimized correlated with an incremental reduction in progression to T2D [112]. There is a growing list of laboratories that are demonstrating the degree to which epigenetically driven aging might be reversed. In addition, it may be possible to combine nutritional and chemical treatments that will reduce risks for chronic diseases including cancers [113]. An “epigenetic diet” has been proposed based on nutrients that affect different epigenetic processes [83].

## 9. Conclusions

It is obvious that the health of the human population is headed in the wrong direction with regard to slowing the epidemic of malnutrition and chronic disease. It is highly unlikely that we will be able to stem the epidemic of chronic disease in the USA without a major change in the food culture in this country. This will require overcoming the major barriers to healthy nutrition for vulnerable people before, during pregnancy, and during lactation and early childhood [114]. The current health trajectory in the US, which is largely the result of poor industrial diets, could lead to a health care crisis in the coming decades.

The more we understand about how stresses like maternal malnutrition affect the long-term disease risks for offspring and human populations, the more discouraged we may become.

There are strategies for improving the nutrition levels of people before they get pregnant, during their pregnancy and lactation, and during early childhood. These include the following:

- Providing higher quality food for public and private schools.
- Teaching the principles of nutrition in schools and universities.
- Training health professionals regarding nutrition as medicine.
- Working with communities to enable nutrition programs for people of reproductive age.
- Enhancing the nutritional offerings for people served by WIC and SNAP.

There is an increasing groundswell of people interested in improving nutrition for people of reproductive age. An increasing number of scientists are applying their expertise to understanding the epigenetic underpinnings of disease. Thus, there is room for some optimism regarding global human health because the awareness of good nutrition is spreading across the land. Readers should contribute their expertise to reverse the poor nutrition that is commonplace among pregnant people in the US.

## References

[1] Mousa A., Naqash A., Lim S. (2019). Macronutrient and Micronutrient Intake during Pregnancy: An Overview of Recent Evidence. Nutrients.

[2] Marshall N.E., Abrams B., Barbour L.A., Catalano P., Christian P., Friedman J.E., Hay W.W., Hernandez T.L., Krebs N.F., Oken E. (2022). The importance of nutrition in pregnancy and lactation: Lifelong consequences. Am. J. Obstet. Gynecol..

[3] Koletzko B., Godfrey K.M., Poston L., Szajewska H., van Goudoever J.B., de Waard M., Brands B., Grivell R.M., Deussen A.R., Dodd J.M. (2019). Nutrition During Pregnancy, Lactation and Early Childhood and its Implications for Maternal and Long-Term Child Health: The Early Nutrition Project Recommendations. Ann. Nutr. Metab..

[4] Bazer F.W., Lamb G.C., Wu G. (2019). Animal Agriculture: Sustainability, Challenges and Innovations.

[5] Reynolds L.P., Ireland J.J., Caton J.S., Bauman D.E., Davis T.A. (2009). Commentary on domestic animals in agricultural and biomedical research: An endangered enterprise. J. Nutr..

[6] Morrison J.L., Regnault T.R. (2016). Nutrition in Pregnancy: Optimising Maternal Diet and Fetal Adaptations to Altered Nutrient Supply. Nutrients.

[7] Dictionary C. Cambridge Dictionary: Epidemic. https://dictionary.cambridge.org/dictionary/english/epidemic.

[8] WHO The True Death Toll of COVID-19: Estimating Global Excess Mortality. https://www.who.int/data/stories/the-true-death-toll-of-covid-19-estimating-global-excess-mortality.

[9] World Health Organization: Noncommunicable Diseases. https://www.who.int/news-room/fact-sheets/detail/noncommunicable-diseases.

[10] Johns Hopkins Medicine Insight. https://www.hopkinsacg.org/how-can-understanding-common-comorbidities-improve-your-population-health-strategy-and-ultimately-patient-outcomes/.

[11] Centers for Medicare & Medicaid Services National Health Expenditure Data. https://www.cms.gov/data-research/statistics-trends-and-reports/national-health-expenditure-data.

[12] Centers for Disease Control and Prevention Diabetes Report Card 2019. https://www.cdc.gov/diabetes/pdfs/library/Diabetes-Report-Card-2019-508.pdf.

[13] Johnson C.Y. The Lingering Health Effects of the Civil War. https://www.washingtonpost.com/news/wonk/wp/2016/01/04/the-lingering-health-effects-of-the-civil-war/.

[14] Barker D.J.P. (2012). Sir Richard Doll Lecture. Developmental origins of chronic disease. Public Health.

[15] Horvath S. (2015). Erratum to: DNA methylation age of human tissues and cell types. Genome Biol..

[16] Gregg E.W., Cadwell B.L., Cheng Y.J., Cowie C.C., Williams D.E., Geiss L., Engelgau M.M., Vinicor F. (2004). Trends in the prevalence and ratio of diagnosed to undiagnosed diabetes according to obesity levels in the U.S. Diabetes Care.

[17] National Diabetes Statistics Report: Estimates of Diabetes and Its Burden in the United States. https://www.cdc.gov/diabetes/php/data-research/index.html.

[18] Deshpande A.D., Harris-Hayes M., Schootman M. (2008). Epidemiology of diabetes and diabetes-related complications. Phys. Ther..

[19] Kim H., Hu E.A., Rebholz C.M. (2019). Ultra-processed food intake and mortality in the USA: Results from the Third National Health and Nutrition Examination Survey (NHANES III, 1988-1994). Public Health Nutr..

[20] Paula W.O., Patriota E.S.O., Goncalves V.S.S., Pizato N. (2022). Maternal Consumption of Ultra-Processed Foods-Rich Diet and Perinatal Outcomes: A Systematic Review and Meta-Analysis. Nutrients.

[21] Fraga A., Bastos M.P., Theme-Filha M.M. (2024). Increased consumption of ultra-processed foods during pregnancy is associated with sociodemographic, behavioral, and obstetric factors: A cohort study. Nutr. Res..

[22] Lourenco B.H., Castro M.C., de Morais Sato P., Neves P.A.R., Vivanco E., Lima D.L., Cardoso M.A., Group M.I.-B.S. (2023). Exposure to ultra-processed foods during pregnancy and ultrasound fetal growth parameters. Br. J. Nutr..

[23] Thornburg K.L., Challis J.R. (2014). How to build a healthy heart from scratch. Adv. Exp. Med. Biol..

[24] Curran M.A. Fetal Development. https://www.perinatology.com/Reference/Fetal%20development.htm#TOP.

[25] Tan C.M.J., Lewandowski A.J. (2020). The Transitional Heart: From Early Embryonic and Fetal Development to Neonatal Life. Fetal Diagn. Ther..

[26] Schittny J.C. (2017). Development of the lung. Cell Tissue Res..

[27] Mahmoud A.I. (2023). Metabolic switches during development and regeneration. Development.

[28] Gernand A.D., Schulze K.J., Stewart C.P., West K.P., Christian P. (2016). Micronutrient deficiencies in pregnancy worldwide: Health effects and prevention. Nat. Rev. Endocrinol..

[29] McCance R.A. (1976). Critical periods of growth. Proc. Nutr. Soc..

[30] Bateson P., Barker D., Clutton-Brock T., Deb D., D’Udine B., Foley R.A., Gluckman P., Godfrey K., Kirkwood T., Lahr M.M. (2004). Developmental plasticity and human health. Nature.

[31] Gluckman P.D., Hanson M.A., Cooper C., Thornburg K.L. (2008). Effect of in utero and early-life conditions on adult health and disease. N. Engl. J. Med..

[32] Luyckx V.A., Bertram J.F., Brenner B.M., Fall C., Hoy W.E., Ozanne S.E., Vikse B.E. (2013). Effect of fetal and child health on kidney development and long-term risk of hypertension and kidney disease. Lancet.

[33] Lawson T.B., Scott-Drechsel D.E., Chivukula V.K., Rugonyi S., Thornburg K.L., Hinds M.T. (2018). Hyperglycemia Alters the Structure and Hemodynamics of the Developing Embryonic Heart. J. Cardiovasc. Dev. Dis..

[34] Simeone R.M., Devine O.J., Marcinkevage J.A., Gilboa S.M., Razzaghi H., Bardenheier B.H., Sharma A.J., Honein M.A. (2015). Diabetes and congenital heart defects: A systematic review, meta-analysis, and modeling project. Am. J. Prev. Med..

[35] Piquereau J., Ventura-Clapier R. (2018). Maturation of Cardiac Energy Metabolism During Perinatal Development. Front. Physiol..

[36] Drake R.R., Louey S., Thornburg K.L. (2023). Maturation of lipid metabolism in the fetal and newborn sheep heart. Am. J. Physiol. Regul. Integr. Comp. Physiol..

[37] Drake R.R., Louey S., Thornburg K.L. (2022). Intrauterine growth restriction elevates circulating acylcarnitines and suppresses fatty acid metabolism genes in the fetal sheep heart. J. Physiol..

[38] National Center for Health Statistics National Health and Nutrition Examination Survey 2017–March 2020 Prepandemic Data Files Development of Files and Prevalence Estimates for Selected Health Outcomes. https://stacks.cdc.gov/view/cdc/106273.

[39] Barker D.J., Thornburg K.L. (2013). The obstetric origins of health for a lifetime. Clin. Obstet. Gynecol..

[40] Barker D.J., Thornburg K.L. (2013). Placental programming of chronic diseases, cancer and lifespan: A review. Placenta.

[41] Fall C.H. (2013). Fetal programming and the risk of noncommunicable disease. Indian. J. Pediatr..

[42] Ricker M.A., Haas W.C. (2017). Anti-Inflammatory Diet in Clinical Practice: A Review. Nutr. Clin. Pract..

[43] Dietary Guidelines for Americans, 2020–2025. https://www.dietaryguidelines.gov/resources/2020-2025-dietary-guidelines-online-materials.

[44] Mozaffarian D., Benjamin E.J., Go A.S., Arnett D.K., Blaha M.J., Cushman M., de Ferranti S., Despres J.P., Fullerton H.J., Howard V.J. (2015). Heart disease and stroke statistics—2015 update: A report from the American Heart Association. Circulation.

[45] Kontogianni M.D., Zampelas A., Tsigos C. (2006). Nutrition and inflammatory load. Ann. N. Y. Acad. Sci..

[46] GBD 2017 Diet Collaborators (2019). Health effects of dietary risks in 195 countries, 1990–2017: A systematic analysis for the Global Burden of Disease Study 2017. Lancet.

[47] Fitzgerald K.N., Hodges R., Hanes D., Stack E., Cheishvili D., Szyf M., Henkel J., Twedt M.W., Giannopoulou D., Herdell J. (2021). Potential reversal of epigenetic age using a diet and lifestyle intervention: A pilot randomized clinical trial. Aging.

[48] Desai M., Ross M.G. (2020). Maternal-infant nutrition and development programming of offspring appetite and obesity. Nutr. Rev..

[49] Buffington S.A., Di Prisco G.V., Auchtung T.A., Ajami N.J., Petrosino J.F., Costa-Mattioli M. (2016). Microbial Reconstitution Reverses Maternal Diet-Induced Social and Synaptic Deficits in Offspring. Cell.

[50] Aurich S., Muller L., Kovacs P., Keller M. (2023). Implication of DNA methylation during lifestyle mediated weight loss. Front Endocrinol.

[51] Yan S.F., Ramasamy R., Schmidt A.M. (2008). Mechanisms of disease: Advanced glycation end-products and their receptor in inflammation and diabetes complications. Nat. Clin. Pract. Endocrinol. Metab..

[52] Kim J., Song G., Wu G., Bazer F.W. (2012). Functional roles of fructose. Proc. Natl. Acad. Sci. USA.

[53] Thompson M.D., DeBosch B.J. (2021). Maternal Fructose Diet-Induced Developmental Programming. Nutrients.

[54] Nakagawa T., Ana A.-H., Kosugi T., Sanchez-Lozada L.G., Stenvinkel P., Kublickiene K., Ananth Karumanchi S., Kang D.H., Kojima H., Rodriguez-Iturbe B. (2023). Fructose might be a clue to the origin of preeclampsia insights from nature and evolution. Hypertens. Res..

[55] Asghar Z.A., Thompson A., Chi M., Cusumano A., Scheaffer S., Al-Hammadi N., Saben J.L., Moley K.H. (2016). Maternal fructose drives placental uric acid production leading to adverse fetal outcomes. Sci. Rep..

[56] Fall C.H. (2013). Fetal malnutrition and long-term outcomes. Nestle Nutr. Inst. Workshop Ser..

[57] Harding J.E. (2001). The nutritional basis of the fetal origins of adult disease. Int. J. Epidemiol..

[58] Bancroft J.S. (1946). Researches on Pre-Natal Life.

[59] Kolahi K.S., Valent A.M., Thornburg K.L. (2017). Cytotrophoblast, Not Syncytiotrophoblast, Dominates Glycolysis and Oxidative Phosphorylation in Human Term Placenta. Sci. Rep..

[60] Burton G.J., Fowden A.L., Thornburg K.L. (2016). Placental Origins of Chronic Disease. Physiol. Rev..

[61] Jansson T., Powell T.L. (2007). Role of the placenta in fetal programming: Underlying mechanisms and potential interventional approaches. Clin. Sci..

[62] Hoffman D.J., Powell T.L., Barrett E.S., Hardy D.B. (2021). Developmental origins of metabolic diseases. Physiol. Rev..

[63] Thornburg K.L., Marshall N. (2015). The placenta is the center of the chronic disease universe. Am. J. Obstet. Gynecol..

[64] Howell K.R., Powell T.L. (2017). Effects of maternal obesity on placental function and fetal development. Reproduction.

[65] Trivett C., Lees Z.J., Freeman D.J. (2021). Adipose tissue function in healthy pregnancy, gestational diabetes mellitus and pre-eclampsia. Eur. J. Clin. Nutr..

[66] Thompson G.N., Halliday D. (1992). Protein turnover in pregnancy. Eur. J. Clin. Nutr..

[67] Duggleby S.L., Jackson A.A. (2001). Relationship of maternal protein turnover and lean body mass during pregnancy and birth length. Clin. Sci..

[68] Jones H.N., Powell T.L., Jansson T. (2007). Regulation of placental nutrient transport—A review. Placenta.

[69] Lager S., Powell T.L. (2012). Regulation of nutrient transport across the placenta. J. Pregnancy.

[70] Brett K.E., Ferraro Z.M., Yockell-Lelievre J., Gruslin A., Adamo K.B. (2014). Maternal-fetal nutrient transport in pregnancy pathologies: The role of the placenta. Int. J. Mol. Sci..

[71] Jansson T., Powell T.L. (2013). Role of placental nutrient sensing in developmental programming. Clin. Obstet. Gynecol..

[72] Myatt L. (2006). Placental adaptive responses and fetal programming. J. Physiol..

[73] Myatt L., Thornburg K.L. (2018). Effects of Prenatal Nutrition and the Role of the Placenta in Health and Disease. Methods Mol. Biol..

[74] Gete D.G., Waller M., Mishra G.D. (2020). Effects of maternal diets on preterm birth and low birth weight: A systematic review. Br. J. Nutr..

[75] Waterland R.A., Jirtle R.L. (2003). Transposable elements: Targets for early nutritional effects on epigenetic gene regulation. Mol. Cell Biol..

[76] Jirtle R.L. (2014). The Agouti mouse: A biosensor for environmental epigenomics studies investigating the developmental origins of health and disease. Epigenomics.

[77] Franzago M., Fraticelli F., Stuppia L., Vitacolonna E. (2019). Nutrigenetics, epigenetics and gestational diabetes: Consequences in mother and child. Epigenetics.

[78] Keleher M.R., Zaidi R., Shah S., Oakley M.E., Pavlatos C., El Idrissi S., Xing X., Li D., Wang T., Cheverud J.M. (2018). Maternal high-fat diet associated with altered gene expression, DNA methylation, and obesity risk in mouse offspring. PLoS ONE.

[79] Mentch S.J., Locasale J.W. (2016). One-carbon metabolism and epigenetics: Understanding the specificity. Ann. N. Y. Acad. Sci..

[80] Smith D.E., Hornstra J.M., Kok R.M., Blom H.J., Smulders Y.M. (2013). Folic acid supplementation does not reduce intracellular homocysteine, and may disturb intracellular one-carbon metabolism. Clin. Chem. Lab. Med..

[81] Thornburg K.L., Shannon J., Thuillier P., Turker M.S. (2010). In utero life and epigenetic predisposition for disease. Adv. Genet..

[82] Perrier F., Viallon V., Ambatipudi S., Ghantous A., Cuenin C., Hernandez-Vargas H., Chajes V., Baglietto L., Matejcic M., Moreno-Macias H. (2019). Association of leukocyte DNA methylation changes with dietary folate and alcohol intake in the EPIC study. Clin. Epigenetics.

[83] Hardy T.M., Tollefsbol T.O. (2011). Epigenetic diet: Impact on the epigenome and cancer. Epigenomics.

[84] Fitz-James M.H., Cavalli G. (2022). Molecular mechanisms of transgenerational epigenetic inheritance. Nat. Rev. Genet..

[85] Liberman N., Wang S.Y., Greer E.L. (2019). Transgenerational epigenetic inheritance: From phenomena to molecular mechanisms. Curr. Opin. Neurobiol..

[86] Aiken C.E., Tarry-Adkins J.L., Ozanne S.E. (2016). Transgenerational effects of maternal diet on metabolic and reproductive ageing. Mamm. Genome.

[87] Desai M., Jellyman J.K., Ross M.G. (2015). Epigenomics, gestational programming and risk of metabolic syndrome. Int. J. Obes..

[88] Serpeloni F., Radtke K., de Assis S.G., Henning F., Natt D., Elbert T. (2017). Grandmaternal stress during pregnancy and DNA methylation of the third generation: An epigenome-wide association study. Transl. Psychiatry.

[89] Roseboom T.J. (2019). Epidemiological evidence for the developmental origins of health and disease: Effects of prenatal undernutrition in humans. J. Endocrinol..

[90] Tobi E.W., Lumey L.H., Talens R.P., Kremer D., Putter H., Stein A.D., Slagboom P.E., Heijmans B.T. (2009). DNA methylation differences after exposure to prenatal famine are common and timing- and sex-specific. Hum. Mol. Genet..

[91] Horsthemke B. (2018). A critical view on transgenerational epigenetic inheritance in humans. Nat. Commun..

[92] Lillycrop K.A., Burdge G.C. (2015). Maternal diet as a modifier of offspring epigenetics. J. Dev. Orig. Health Dis..

[93] Morgan C.P., Bale T.L. (2017). Sex differences in microRNA-mRNA networks: Examination of novel epigenetic programming mechanisms in the sexually dimorphic neonatal hypothalamus. Biol. Sex. Differ..

[94] Zhu Y., Olsen S.F., Mendola P., Halldorsson T.I., Yeung E.H., Granstrom C., Bjerregaard A.A., Wu J., Rawal S., Chavarro J.E. (2017). Maternal dietary intakes of refined grains during pregnancy and growth through the first 7 y of life among children born to women with gestational diabetes. Am. J. Clin. Nutr..

[95] Godfrey K.M., Sheppard A., Gluckman P.D., Lillycrop K.A., Burdge G.C., McLean C., Rodford J., Slater-Jefferies J.L., Garratt E., Crozier S.R. (2011). Epigenetic gene promoter methylation at birth is associated with child’s later adiposity. Diabetes.

[96] Park L.K., Friso S., Choi S.W. (2012). Nutritional influences on epigenetics and age-related disease. Proc. Nutr. Soc..

[97] Economic Research Service U.S. Department of Agriculture, Agriculture and Food Statistics Charting the Essentials. https://www.ers.usda.gov/data-products/ag-and-food-statistics-charting-the-essentials.

[98] Moss M. (2013). Salt Sugar Fat: How the Food Giants Hookes Us.

[99] Fuhrman J. (2018). The Hidden Dangers of Fast and Processed Food. Am. J. Lifestyle Med..

[100] Bayol S.A., Macharia R., Farrington S.J., Simbi B.H., Stickland N.C. (2009). Evidence that a maternal “junk food” diet during pregnancy and lactation can reduce muscle force in offspring. Eur. J. Nutr..

[101] Bayol S.A., Simbi B.H., Fowkes R.C., Stickland N.C. (2010). A maternal “junk food” diet in pregnancy and lactation promotes nonalcoholic Fatty liver disease in rat offspring. Endocrinology.

[102] Smith B.L., Reyes T.M. (2017). Offspring neuroimmune consequences of maternal malnutrition: Potential mechanism for behavioral impairments that underlie metabolic and neurodevelopmental disorders. Front. Neuroendocrinol..

[103] Shaw G.M., Yang W. (2019). Women’s periconceptional lowered carbohydrate intake and NTD-affected pregnancy risk in the era of prefortification with folic acid. Birth Defects Res..

[104] Gardner C.D., Vadiveloo M.K., Petersen K.S., Anderson C.A.M., Springfield S., Van Horn L., Khera A., Lamendola C., Mayo S.M., Joseph J.J. (2023). Popular Dietary Patterns: Alignment With American Heart Association 2021 Dietary Guidance: A Scientific Statement From the American Heart Association. Circulation.

[105] Mambrini S.P., Menichetti F., Ravella S., Pellizzari M., De Amicis R., Foppiani A., Battezzati A., Bertoli S., Leone A. (2023). Ultra-Processed Food Consumption and Incidence of Obesity and Cardiometabolic Risk Factors in Adults: A Systematic Review of Prospective Studies. Nutrients.

[106] Gohir W., Kennedy K.M., Wallace J.G., Saoi M., Bellissimo C.J., Britz-McKibbin P., Petrik J.J., Surette M.G., Sloboda D.M. (2019). High-fat diet intake modulates maternal intestinal adaptations to pregnancy and results in placental hypoxia, as well as altered fetal gut barrier proteins and immune markers. J. Physiol..

[107] McCullough L.E., Miller E.E., Calderwood L.E., Shivappa N., Steck S.E., Forman M.R., M A.M., Maguire R., Fuemmeler B.F., Kollins S.H. (2017). Maternal inflammatory diet and adverse pregnancy outcomes: Circulating cytokines and genomic imprinting as potential regulators?. Epigenetics.

[108] Zavalza-Gomez A.B., Anaya-Prado R., Rincon-Sanchez A.R., Mora-Martinez J.M. (2008). Adipokines and insulin resistance during pregnancy. Diabetes Res. Clin. Pract..

[109] Aye I.L., Powell T.L., Jansson T. (2013). Review: Adiponectin—The missing link between maternal adiposity, placental transport and fetal growth?. Placenta.

[110] Weaver I.C., Champagne F.A., Brown S.E., Dymov S., Sharma S., Meaney M.J., Szyf M. (2005). Reversal of maternal programming of stress responses in adult offspring through methyl supplementation: Altering epigenetic marking later in life. J. Neurosci..

[111] Mazzio E.A., Soliman K.F. (2014). Epigenetics and nutritional environmental signals. Integr. Comp. Biol..

[112] Yang J., Qian F., Chavarro J.E., Ley S.H., Tobias D.K., Yeung E., Hinkle S.N., Bao W., Li M., Liu A. (2022). Modifiable risk factors and long term risk of type 2 diabetes among individuals with a history of gestational diabetes mellitus: Prospective cohort study. BMJ.

[113] Tiffon C. (2018). The Impact of Nutrition and Environmental Epigenetics on Human Health and Disease. Int. J. Mol. Sci..

[114] Rainford M., Barbour L.A., Birch D., Catalano P., Daniels E., Gremont C., Marshall N.E., Wharton K., Thornburg K. (2024). Barriers to implementing good nutrition in pregnancy and early childhood: Creating equitable national solutions. Ann. N. Y. Acad. Sci..

